# The Association Between Vitamin D and Multiple Sclerosis Risk: 1,25(OH)_2_D_3_ Induces Super-Enhancers Bound by VDR

**DOI:** 10.3389/fimmu.2019.00488

**Published:** 2019-03-19

**Authors:** Ming Lu, Bennet J. McComish, Kathryn P. Burdon, Bruce V. Taylor, Heinrich Körner

**Affiliations:** ^1^Menzies Institute for Medical Research, University of Tasmania, Hobart, TAS, Australia; ^2^Department of Immunology, Anhui Medical University, Hefei, China; ^3^Key Laboratory of Anti-inflammatory and Immunopharmacology, Ministry of Education, Collaborative Innovation Center of Anti-Inflammatory and Immune Medicine, Institute of Clinical Pharmacology, Anhui Medical University, Hefei, China

**Keywords:** vitamin D, vitamin D receptor, inducible super-enhancer, risk allele, multiple sclerosis

## Abstract

A super-enhancer (SE) is a cluster of enhancers with a relatively high density of particular chromatin features. SEs typically regulate key genes that can determine cell identity and differentiation. Identifying SEs and their effects may be critical in predicting key regulatory genes, such as master transcription factor genes or oncogenes. Signal inducible SEs are dense stretches of signal terminal transcription factor (TF) binding regions, and may modulate the interaction between environmental factors (e.g., Vitamin D) and genetic factors (i.e., risk variants) in complex diseases such as multiple sclerosis (MS). As a complex autoimmune disease, the etiology and progression of MS, including the interaction between Vitamin D and MS risk variants, is still unclear and can be explored from the aspect of signal SEs. Vitamin D [with its active form: 1,25(OH)_2_D_3_], is an environmental risk factor for MS. It binds the Vitamin D receptor (VDR) and regulates gene expression. This study explores the association between VDR super-enhancers (VSEs) and MS risk variants. Firstly, we reanalyse public ChIP-seq and RNA-seq data to classify VSEs into three categories according to their combinations of persistent and secondary VDR binding. Secondly, we indicate the genes with VSE regions that are near MS risk variants. Furthermore, we find that MS risk variants are enriched in VSE regions, and we indicate some genes with a VSE overlapping MS risk variant for further exploration. We also find two clusters of genes from the set of genes showing correlation of expression patterns with the MS risk gene *ZMIZ1* that appear to be regulated by VSEs in THP-1 cells. It is the first time that VSEs have been analyzed, and we directly connect the genetic risk factors for MS risk with Vitamin D based on VSEs.

## Introduction

The transcription of genes depends on the interaction of their promoter regions with polymerases that synthesize RNA from genomic DNA in combination with an array of regulatory factors (1, 2). Additionally, transcription is aided by cis-acting regulatory elements that can be located relatively distant to the promoter region and can bind activator proteins. These enhancer elements are able to increase the level of gene transcription.

Super-enhancers (SEs) are dense clusters of enhancers. They differ from typical enhancers (TEs) in the density of enhancer elements. Enhancers, including both TEs and SEs, can be annotated by histone status, i.e., H3K27ac and H3K4me1. In addition, high chromatin accessibility [e.g., as determined using FAIRE-seq or DNaseI hypersensitive sites (DHS)], master transcription factor binding (e.g., PU.1 for monocytes, RORγt for Th17 cells), and pervasive factors in the transcription machinery (e.g., p300, MED1, BRD4, and RNA polymerase II) are all highly correlated with SE regions and can be used to identify SE regions.

Although defined arbitrarily according to enhancer signal density, SEs have proved extremely valuable in predicting key regulatory regions or genes for cell identity or cell differentiation (3). For example, inappropriate acquisition of SE in an oncogene, such as *c-MYC*, will increase its expression and lead to oncogenesis (4, 5). SE regions promote the expression of autoimmune disease-associated genes. For example, the drug JQ1 [BET (bromodomain and extra-terminal domain) inhibitor] inhibits the expression of inflammatory arthritis risk gene *CXCR4* by affecting its SE region (6), and tofacitinib [JAK (Janus kinase) inhibitor] disproportionately inhibits the expression of rheumatoid arthritis risk genes with SE regions compared with those risk genes without SE regions (7).

Previous research has focused on classic SEs identified by chromatin accessibility, master transcription factors or pervasive factors in the transcription machinery, but recently, a new concept of signal-inducible SEs has been proposed (8). It was found that estrogen could induce the generation of new signal SE regions, which were bound by the terminal transcription factor (TF) ERα of the estrogen signaling pathway. The advantage of signal SEs for research is that they can provide important information on the signal terminal TF cistrome before and after signal stimulation.

Multiple sclerosis (MS) is a complex autoimmune disease with multiple risk factors including genetic variants and Vitamin D deficiency (9, 10). Until now, the functional variants of many genome-wide association study (GWAS) loci have not been identified. In addition, the mechanism underlying the interaction between genetic factors and Vitamin D in MS etiology and progression is still unclear. Some MS risk single nucleotide polymorphisms (SNPs) have been found located in Vitamin D Receptor (VDR) binding sites in lymphoblastoid cell lines (LCLs) (11) and conversely, VDR binding sites are also enriched in MS risk regions identified by GWAS (MS risk SNP ± 100 kb) (12). Furthermore, MS risk SNPs are enriched in classic SE regions of CD4^+^ T cells and monocytes (3, 7). The risk alleles could potentially modulate the regulatory effects of these SEs on key genes in specific cell types.

We hypothesize that VDR super-enhancers (VSEs) may be signal inducible SEs relevant to MS development, and that GWAS-identified MS risk loci may influence the function of such VSEs. To assess this, we re-analyzed next-generation sequencing (NGS) data from cell stimulation experiments employing hormones and their nuclear receptors. In particular, we were interested in the 1,25(OH)_2_D_3_ (the active form of Vitamin D) and VDR couple, and its association with MS. Firstly, we analyzed the overlap between VSEs and classic SEs on their genomic regions and closest genes. We classified all VSEs into three patterns (VSE1: only persistent VDR binding; VSE2: both persistent and secondary VDR binding; VSE3: only secondary VDR binding) and analyzed their characteristics. Furthermore, we analyzed the enrichment of MS risk SNPs in VSE regions, and confirmed that VSEs were significantly enriched for MS risk SNPs.

*ZMIZ1* and *EOMES* have been identified as MS risk genes by cohort studies, and are differentially expressed in whole blood between MS patients and healthy controls (13–15). *ZMIZ1* is highly expressed in monocytes and *EOMES* is predominantly expressed in NK cells. *ZMIZ1* is known to regulate the activity of various transcription factors. *ZMIZ1* and a set of genes with a correlated expression pattern are under-expressed in blood of MS patients (15). We identified two gene clusters in the *ZMIZ1*-correlated gene set, one with high response to 1,25(OH)_2_D_3_ and the other with high expression levels in THP-1 cells, that are potentially affected by VSE2 regions and VSE3 regions, respectively.

Our research shows an association between VDR super-enhancer regions and MS risk for the first time.

## Materials and Methods

### Next Generation Sequencing Data Selection

We downloaded unstimulated and 1,25(OH)_2_D_3_-stimulated VDR ChIP-seq, PU.1 ChIP-seq, FAIRE-seq, and RNA-seq data in THP-1 cells, and other hormone/nuclear receptor (i.e., estrogen/ERα and dexamethasone/GR) NGS data (SRA format), from the NCBI Gene Expression Omnibus (GEO) database (Table 1 and Table S1). Then FASTQ files were converted from the SRA files with command “fastq-dump.”

**Table 1:** Control and 1,25(OH)_2_D_3_ (D3)-stimulated ChIP-seq, FAIRE-seq, and RNA-seq data in THP-1 cells (VDR_1).

**Transcription factor**	**Cell type**	**Signal**	**Treatment**	**Organism**	**Vehicle**	**Hormone**	**References**
VDR	THP-1	D3	100 nM 24 h	human	GSM2371448	GSM2371449	(16)
					SRR4828896	SRR4828897	
PU.1	THP-1	D3	100 nM 24 h	human	GSM2359982	GSM2359983	(17)
					SRR4450164	SRR4450165	
Faire-seq	THP-1	D3	100 nM 24 h	human	GSM1697277(0h)	GSM1697279	(18)
					SRR2042800	SRR2042802	
RNA-seq	THP-1	D3	100 nM 24 h	human	GSM1697100,06,12	GSM1697101,07,13	(18)
					SRR2042584,90,96	SRR2042585,91,67	
Input					GSM1714041		(18)
					SRR2067928		

### ChIP-seq Pipeline

Bowtie2 was used to align FASTQ files to the hg19 reference genome with bowtie2 indexes (“bowtie2 -x -U -S”) (19). We defined peaks with MACS2 (20). For ChIP-seq data from transcription factors VDR and PU.1, we used the command “macs2 callpeak –bw 150 –keep-dup 1 -q 0.01 –B.” For FAIRE-seq, we used a nomodel command “macs2 callpeak –nomodel –shift−75 –extsize 150 –keep-dup 1 -q 0.01 –B.” Potential artifact signals, based on publicly available blacklists of genomic regions known to have anomalous, unstructured, high signal/read counts in next gen sequencing experiments (21), were removed from the peak sets using “BEDTools intersect” (22).

### Identification and Classification of Super-Enhancers

We distinguished SEs from TEs using ROSE with command setting “-t 2000 -s 12500” (4, 23). Firstly, promoter regions [i.e., 2 kb upstream and downstream of the transcription start site (TSS)] with ChIP-seq or FAIRE-seq annotations were excluded. Then enhancer regions identified by the chosen enhancer annotation were stitched together within defined regions of length 12.5 kb to generate signal densities and ranked in order of enhancer density. On a plot of signal density vs. density rank the tangent point is identified by a tangent line with a slope of 1 and divides the enhancers into two types: SEs (with higher density, to the left of the tangent point) and typical enhancers (TEs with lower density, to the right of the tangent point) (3, 4, 23).

To count the signal density for density correlation analysis, reads were extended by 200 bp and the density of reads per base pair was calculated using bamToGFF (https://github.com/BradnerLab/pipeline), which ROSE integrates internally for identifying SEs (3, 23). These densities were normalized in units of reads per million mapped reads per base pair (rpm/bp) with background subtraction for density correlation analysis between different transcription factors. Integrative Genomics Viewer (IGV) was used to visualize genomic signal density (24).

To classify VSE regions, we firstly defined primary (existing only before signal stimulation), persistent (existing both before and after signal stimulation) and secondary (existing only after signal stimulation) VDR binding sites by comparing VDR binding sites before and after 1,25(OH)_2_D_3_ stimulation. We then selected VSEs after signal stimulation that overlap with persistent VDR binding only (classified as VSE1), secondary VDR binding only (classified as VSE3), or both (classified as VSE2). We also selected VSEs before signal stimulation that overlap with persistent VDR binding only (classified as VSE4), primary VDR binding only (classified as VSE6), or both (classified as VSE5). “BEDTools intersect” (22) was used to classify VSE regions by intersecting VSEs with the different types of VDR binding sites.

### Assigning a Gene to Its Closest Super-Enhancer

We assigned all genes with GENCODE hg19 gene/lncRNA annotations to their closest SEs within a 50 kb window using “BEDTools closest” (22). This window size can identify most true enhancer/promoter interactions as reported in previous studies (3, 23, 25, 26). We used the Venny 2.1 online tool (http://bioinfogp.cnb.csic.es/tools/venny/) (27) to generate Venn diagrams of the interactions between gene sets.

### MS Risk SNPs

MS risk SNPs were downloaded and merged from both NHGRI GWAS Catalog data (https://www.ebi.ac.uk/gwas/search?query=MULTIPLE%20SCLEROSIS) and the most recent International MS Genetics Consortium (IMSGC) results (BioRxiv: https://doi.org/10.1101/143933). SNPs in linkage disequilibrium with the list of MS risk tag SNPs were determined from both HapMap3 and the 1000 Genomes Project using *r*^2^ > 0.8, distance limit = 500 kb and population CEU (Center d'Etude du Polymorphisme Humain–Utah) using the SNP Annotation and Proxy Search (SNAP) tool (https://www.broadinstitute.org/snap/snap) (28).

### Enrichment Analysis in Super-Enhancer Regions

Enrichment levels of TF binding sites or SNPs in SE regions were analyzed via permutation test using the “region” R package, with z-score as a measure of the strength of the association that is independent of the number of permutations (29). As z-score is defined as the distance between the expected value and the observed one, measured in standard deviations, we use z-score 1.96 as a significance test for two sided *p* < 0.05. Enquiry regions (e.g., SNP regions), were normalized by dividing them by the total size of the regions of interest (VSE: 24,014,217 bp, VSE1: 82,635 bp, VSE2: 1,280,936 bp, VSE3: 1,040,646 bp, VDR typical enhancer (VTE): 5,787,494 bp, PU.1 identified SE (PSE): 5,496,182 bp, FAIRE-seq identified SE (FSE): 1,182,725 bp) and reporting them in every 10 Mb of the genome as described in (7). The permutation test for the enrichment *p*-value was performed by generating 1,000 permutations of regions of interest (VSE, VTE, PSE, FSE) in the genome (excluding blacklisted regions and the region of interest itself in each permutation) and considering the total size-normalized number (number per 10 Mb) of overlaps between the enquiry region and the region of interest.

For the SNPs enriched in SE regions, we used RegulomeDB to identify DNA features and regulatory elements such as eQTLs, chromatin signatures and transcription factor binding sites overlapping with these SNPs (30). RegulomeDB scores these SNPs according to the strength of their risk evidence. We report only the SNP with the top score in each SE region.

### Analysis of Super-Enhancer Region Characteristics

We performed gene ontology analysis on super-enhancer regions using the Genomic Regions Enrichment of Annotations Tool (GREAT version 3.0.0) with the whole genome as background and default parameters (31). Pearson's product moment correlation coefficient was used to test for association between paired TF binding densities with value log_2_(rpm/bp) at the same regions. The “ggpubr” R package (https://CRAN.R-project.org/package=ggpubr) was used to produce correlation plots. The *p*-value for correlation was corrected by Bonferroni correction. “BEDTools nuc” (22) was used to count GC content in genomic regions.

### RNA-seq Pipeline to Determine Genes Regulated by 1,25(OH)_2_D_3_

Hisat2 was used to align sequences (three replicates of RNA-seq data) to the hg19 reference genome with Hisat2 indexes (32). The resulting BAM files were sorted by read name, then Htseq was used to count reads on exons in GENCODE hg19 gene annotations with the command “htseq-count -s no -m intersection-nonempty -i gene_name” (33). We only retained genes expressed at a counts-per-million [CPM: calculated using the function cpm from the edgeR library (34) in R] >0.5 in at least two samples. The function “voom” from the limma R package was used to find differentially expressed genes, and “treat” was used to correct the voom results relative to a false discovery rate of 5%. Genes with *p* < 0.05 and log_2_(fold change) ≥1 were considered to be significantly regulated.

The “dunn.test” R package (https://CRAN.R-project.org/package=dunn.test) was used for multiple pairwise comparisons with Bonferroni correction after a Kruskal–Wallis test on the expression level or the regulatory effect between different VDR binding patterns. Violin plots, boxplots, SE curve and transcriptome plots were generated using the “ggplot2” R package (35).

### *ZMIZ1* Gene Set

To generate the gene set that is positively associated with the expression of MS risk gene *ZMIZ1* in whole blood, we merged the top 200 genes [data from (14, 15)] that are positively correlated with *ZMIZ1* expression from each of three cohorts: ANZgene (microarray) cohort (36), Sydney RNASeq cohort (37), clinically isolated syndrome (CIS) cohort (38).

### Gene Set Enrichment Analysis

Analyses focused only on genes under the significant regulation of 1,25(OH)_2_D_3_ (*p* < 0.05 and log_2_(fold change) ≥1) or with a high expression level (average log_2_(expression) >5) ignore the genes that have a relatively small change or a low expression level, which can nevertheless function in a coordinated way in a set of related genes. Gene set enrichment analysis (GSEA) (39, 40) addresses this limitation and was used to find the enrichment distribution of a specific gene set (e.g., VSE gene sets) in a certain pre-defined and pre-ranked gene set (e.g., *ZMIZ1* gene set ranked by log_2_(fold change) or by average log_2_(expression) from high to low), which can indicate a cluster of enriched important genes with a relative high change even though their log_2_(fold change) < 1, or with a high expression level even though their average log_2_(expression) ≤5.

### Gene Ontology Analysis on Gene Clusters

Gene ontology (GO) biological process (BP) terms and Kyoto Encyclopedia of Genes and Genomes (KEGG) pathways are used to annotate the function of *ZMIZ1*-associated VSE gene clusters via “clusterProfiler” R package (41). For GO biological process (BP) analysis, we report the top six significant biological process terms with *p* < 0.05 and *q* < 0.01.

## Results

### The Difference Between Signal SEs and Classic SEs

As the VDR is the only receptor and terminal TF for the signal molecule 1,25(OH)_2_D_3_ (the active form of vitamin D), the cistrome, especially the SE region, of the active VDR reflects the genomic effect of 1,25(OH)_2_D_3_. For this study, we used NGS data from THP-1 cells (human monocytic cell line derived from an acute monocytic leukemia patient) before and after 1,25(OH)_2_D_3_ stimulation (Table 1).

**Figure 1:**
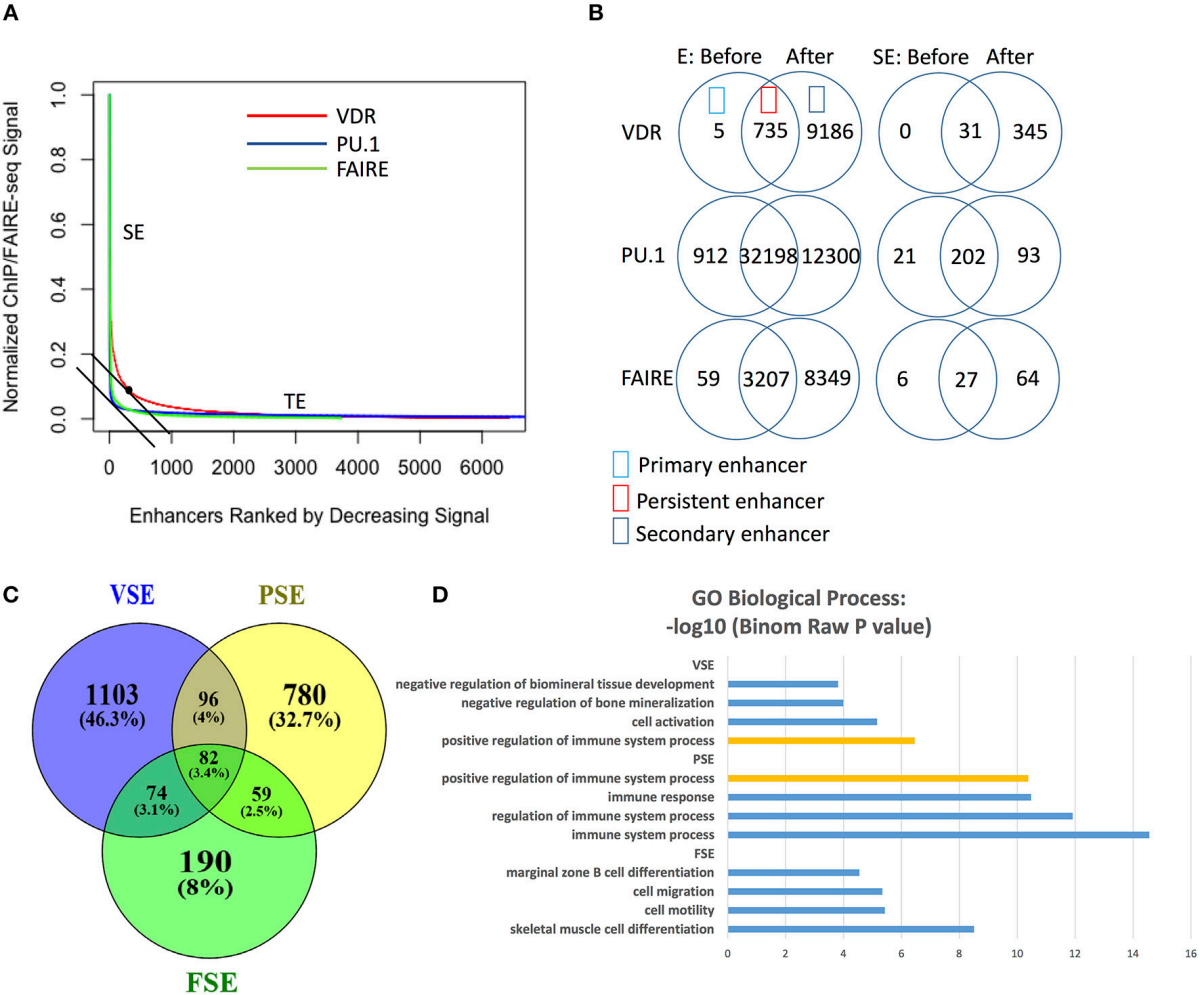
VDR, PU.1, FAIRE identify different SE regions with different genes nearby and gene function enrichment, respectively. **(A)** The (signal density)/(density rank) curve for SE calling from VDR, PU.1 and FARIE. **(B)** The number of enhancers (E) and super-enhancers (SE) before and after 1,25(OH)_2_D_3_ stimulation. **(C)** The Venn overlap figure for the gene list near VSE, PSE and FSE in 50 kb regions. VSE, VDR SE; PSE, PU.1 SE; FSE, FAIRE SE. **(D)** The enrichment analysis of GO biological process gene set for VSE, PSE and FSE; orange bar: the shared process between VSE and PSE (GEO data shown in Table 1).

To explore the difference between signal SEs (e.g., those identified by VDR binding) and classic SEs (e.g., those identified by master TF binding, H3K27ac, or accessible chromatin regions), we called SE regions using data from VDR ChIP-seq, PU.1 ChIP-seq (master TF binding regions), and FAIRE-seq (chromatin open regions) (Supplementary File 1). We found that both PU.1 and FAIRE-seq provided a much clearer distinction between SEs and TEs on the density curve than VDR did (Figure 1A). VDR binding regions achieved higher signal densities and were identified as SE regions at lower ranks (higher rank numbers) (Figure 1A) compared to PU.1-identified SEs (PSEs) and FAIRE-seq-identified SEs (FSEs). As well as higher signal density, the number of VDR enhancers also increased strongly after 1,25(OH)_2_D_3_ stimulation (Figure 1B). Although the numbers of enhancers identified by PU.1 ChIP-seq and FAIRE-seq also increased greatly after stimulation, the numbers of their SEs increased much less than the number of VSEs (Figure 1B). These results reflect the higher sensitivity of signal SE formation in response to signal stimulation compared with that of classic SE formation.

The VSE gene set (the closest genes within 50 kb around VSEs) shares only minor overlap with the PSE and FSE gene sets (Figure 1C). Gene ontology (GO) analysis on super-enhancer regions consistently shows that VSE, PSE, and FSE have different functional gene enrichment, except that the “positive regulation of immune system process” gene set is enriched in both VSE (Supplementary File 1 and Figure 1D) and PSE (Figure 1D). From the GO analysis (Figure 1D), only PSE genes are all enriched in immune associated processes. FSE genes are more often related to other functions, such as “cell mobility” and “cell migration,” and VSE genes are more clearly associated with “cell activation” and “bone mineralization” (Figure 1D).

### Classification of VDR Super-Enhancers

**Figure 2:**
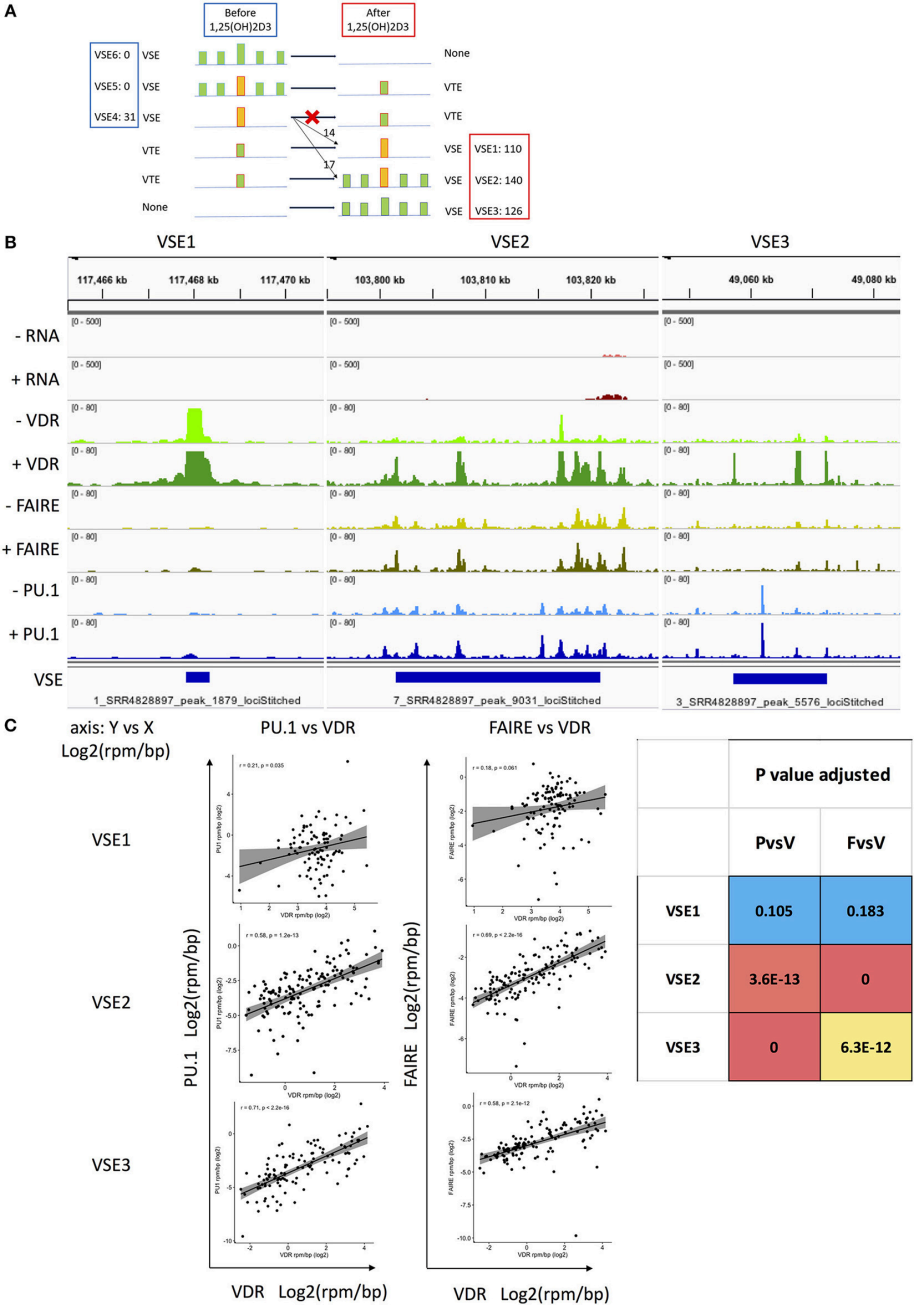
The classification of VSEs and their association with the region and density of PU.1 and FAIRE. **(A)** Classification hypothesis: six types of VSE pattern. VSE4–6 exist without 1,25(OH)_2_D_3_ treatment. VSE1–3 are gained after 1,25(OH)_2_D_3_ treatment. VTE, VDR typical enhancer; VSE, VDR SE; PSE, PU.1 SE; FSE, FAIRE SE; yellow bar, high read density in narrow region identified as SE; read density of bar, yellow> green; blue line, genomic region; The colon of VSE1–6 followed by the number of locations observed that match the pattern in THP-1 cells. **(B)** The representative genomic binding density of VDR, FAIRE, and PU.1 in VSE1–3. **(C)** The signal density correlation between PU.1, VDR and FAIRE in VSE1–3 regions.

As classic SEs appear to play a minor role in genomic responses to signal given their small increase in numbers after signal stimulation, VSEs should potentially play a greater role in 1,25(OH)_2_D_3_ stimulated THP-1 cells. By analyzing the data from THP-1 cells, we found that there were numerous signal VSE regions with persistent VDR binding only (classified as VSE1, *n* = 126), or with secondary VDR binding only (classified as VSE3, *n* = 110), and an even greater number with both persistent and secondary binding (classified as VSE2, *n* = 140) (Figure 2A and Supplementary File 1).

In undertaking these analyses, we assumed that all six potential patterns of VSE were possible (i.e., primary binding, secondary binding or both, before and after 1,25(OH)_2_D_3_ stimulation). Three patterns (VSE4–6) lose their VSE status after treatment and three patterns (VSE1–3) gain VSE status after treatment (Figure 2A). However, we found no evidence for VSE5 or VSE6 existing, and none of the 31 VSE4 regions degenerated into typical enhancers after stimulation. Of the VSE4 regions, 14 were persistent as VSE1 after stimulation, and 17 VSE4 regions initiated secondary VDR binding around them reclassifying as VSE2 after stimulation (Figure 2A). We therefore classify the signal SEs after stimulation into three types: VSE1, VSE2, and VSE3 (Figures 2A,B). Among them, the densities of PU.1/FAIRE-seq and VDR are correlated with each other in VSE2 and VSE3 regions, while the densities of VDR in VSE1 are too high to be correlated with either PU.1 binding density or FAIRE-seq density (Figure 2C).

A previous study found that persistent VDR binding sites have more canonical motif enrichment. Further, different combinations of persistent and transient VDR binding sites in topologically associating domains (TADs) regulate different biological processes (16). Another previous study found that the persistent signal enhancer (ERα binding) initiates the whole signal SE (ERα SE) region by promoting the activation of secondary enhancers (ERα binding) around it after estrogen stimulation, which is the same pattern seen for VSE2 in our study (Figure 2A) (8). To explore the reason the previous study on ERα SEs did not find ERα SE1 and SE3 patterns, but only SE2 (i.e., persistent ERα binding initiates a long ERα SE region), we analyzed ChIP-seq data for two other nuclear receptors (ERα and glucocorticoid receptor) in other cell types. We found that the different signals have different distributions of their signal SE1–3s (Table 2, data set shown in Table S1) (42–46), confirming the signal specificity for the proportions of signal SE1–3 patterns. The major pattern of ERα SEs is SE2 with only a few SE1 and SE3, which explains why previous reports only provides one model for signal SEs (i.e., only the SE2 pattern) (8). For dexamethasone stimulation, the major pattern of glucocorticoid receptor (GR) SEs is GR SE3 with a few SE1 and SE2 in either human B cell type (RS4;11) or mouse macrophage (BMDM).

**Table 2:** The number of signal SE 1–3 for different signals and in different cell types.

**Cell type**		**THP-1**	**THP-1**	**MCF-7**	**MCF-7**	**RS4;11**	BMDM^*^
Corresponding data (Table 1 and Table S1)		VDR_1	VDR_2	ER_1	ER_2	GR_1	GR_2
Signal stimulation		D3	D3	E2	E2	dex	dex; LPS
Transcription factor		VDR	VDR	ERα	ERα	GR	GR
Treatment		100 nM 24 h	10 nM 40 min	100 nM 1 h	100 nM 45 min	10 nM 1 h	100 nM 45 min; 10 ng/ml 45 min
After stimulation	SE1	110	41	17	43	0	1
	SE2	140	6	**151**	**639**	0	4
	SE3	126	19	21	0	**623**	**67**

*D3, 1,25(OH)_2_D_3_; E2, estradiol; dex, dexamethasone; LPS, lipopolysaccharides*.

* *Using mm10 reference genome*.

*Bold values: the number of the major SE type*.

### The Regulatory Effects of VSEs on the Expression of MS Associated Genes

**Figure 3:**
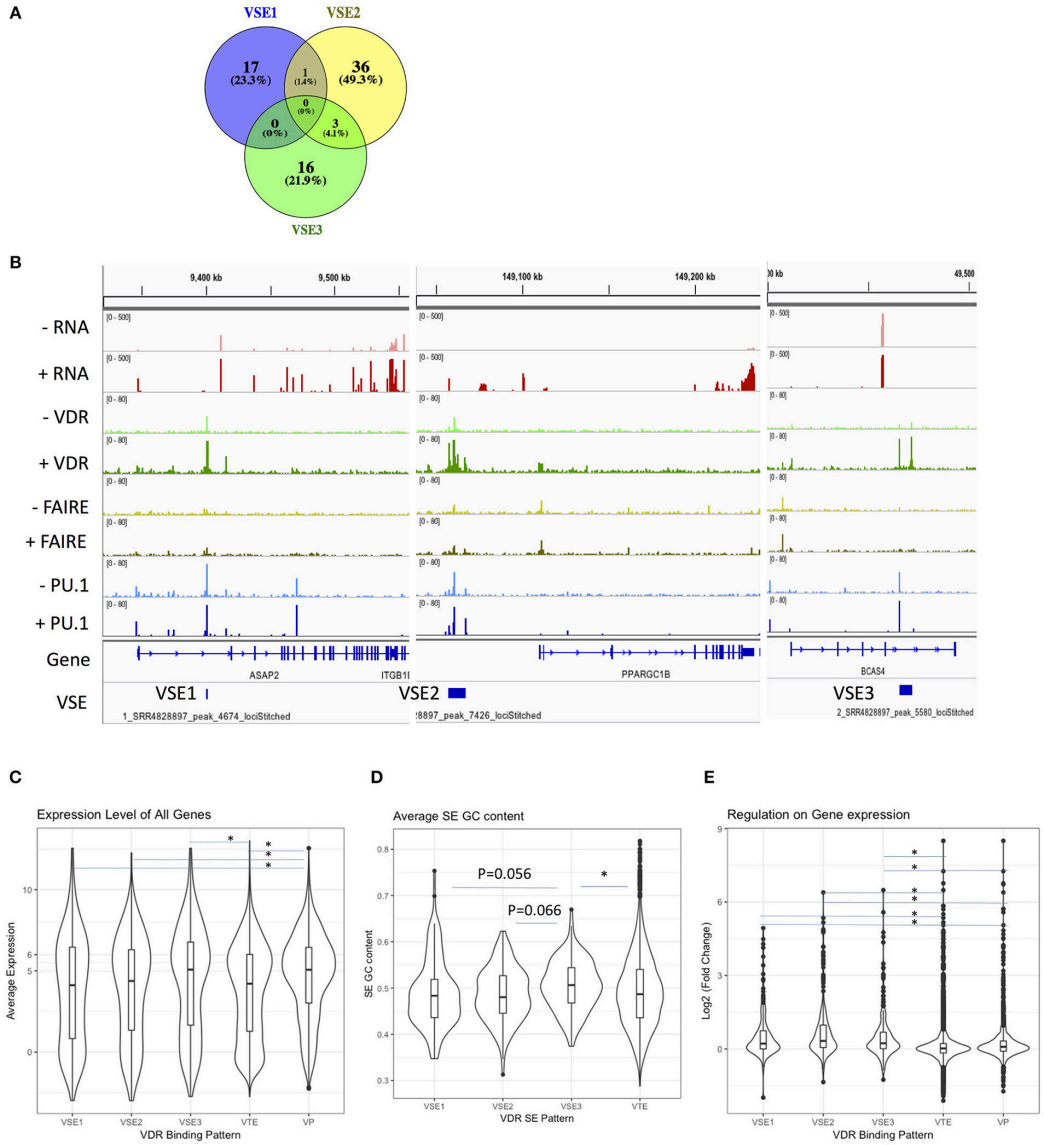
The regulatory effects of VSE1–3 on gene expression. **(A)** The overlap between VSE1–3 close genes. **(B)** The visualization of signal density in representative VSE1–3 regions. **(C)** Average expression level of genes close to different regulatory patterns. **(D)** The GC content of VSE and VTE. **(E)** Regulatory effect of different regulatory patterns on gene expressions. VSE, VDR SE; VTE, VDR typical enhancer; VP, VDR promoter binding. To analyse statistical significance, we used a Kruskal–Wallis test followed by Bonferroni correction ^*^p < 0.05.

To explore the regulatory effect of VSEs on MS gene expression, we investigated all 202 genes that are significantly regulated by 1,25(OH)_2_D_3_ stimulation [with *p* < 0.05 and log_2_(fold change) ≥1] (data set shown in Table 1). The VSE regions account for 73 of the 202 differentially expressed genes and there is little overlap between the gene sets regulated by the three classes of VSEs (Figure 3A). All 73 VSE-regulated genes are shown in Figure S1, with representative genes shown in Figure 3B.

As the high level of average expression [i.e., average log_2_(expression) >5] indicates a basic high chromatin accessibility, we tested the average expression level of VSE1–3 gene sets. We found that genes with VDR binding in the promoter region have higher average expression levels than all other VDR binding patterns except VSE3 (Figure 3C). Both the gene sets of VSE3 and VDR promoter binding, but not VSE1 and VSE2, have a higher average expression level than the VDR TE gene set (Figure 3C). Consistently, we found that among VSE1–3, only VSE3 has a significantly higher GC content than VDR TE, and it has a modestly, although not significantly, higher GC content than VSE1 and VSE2 (Figure 3D), suggesting a more active regulatory conformation and more frequent TF binding (BioRxiv: http://dx.doi.org/10.1101/105262).

However, high expression does not mean a stronger regulatory effect. We found that all VSE1–3 regions have a significantly stronger regulatory effect on gene expression than VDR promoter binding (Figure 3E). VDR promoter binding in turn has a significantly stronger regulatory effect than VDR TE (Figure 3E).

We identified MS risk regions detected by GWAS (MS risk SNP ± 500 kb) that overlap with VSEs, and further evaluated the expression levels of genes in these regions in response to 1,25(OH)_2_D_3_ exposure in THP-1 cells (Table 3 and Figure S2). Among them, genes *DENND6B, USP2, ASAP2, SEMA6B*, and *LRG1* not only have VSE regions and are near MS risk SNPs, but also are significantly regulated by 1,25(OH)_2_D_3_ and highly expressed in THP-1 monocytes. Interestingly, few highly expressed genes with VSE3 patterns are significantly regulated by 1,25(OH)_2_D_3_.

**Table 3:** The genes with VSEs that are associated with MS risk SNPs.

**Genes with VSE that within 500 kb of MS risk SNPs**	**Genes under significant regulation (Log_**2**_FC > 1 and *p* < 0.05)**	**Genes with high expression level (Log_**2**_AverExp > 5)**	**Both of above**
with VSE1	*DENND6B PDCD1LG2 USP2 ASAP2 USP2-AS1 AC080112.2*	*DENND6B USP2 ASAP2 AIG1 VPS37B PLXNB2 RNF26 YPEL3 ALDOA PPP4C RARA PPP6R2 ZC3HAV1 TOP2A*	***DENND6B USP2 ASAP2***
with VSE2	*IGLV7-46 SEMA6B NR1I2 LRG1*	*SEMA6B LRG1 MYO9B CYTH4 BCL9L ELL MUC1 LCP2 CLCN7 TOP1 TELO2 ZMIZ1 COLGALT1 SLC27A1 RAC2 NR2F6 GSE1 PGLS SLC50A1 FKBP8 IRF8 UBASH3B DPM3 GBA GSK3B UBAC2 THBS3*	***SEMA6B LRG1***
with VSE3	*LINC00917 AC023590.1 LRRC25*	*ELL CUEDC1 IRF5 SLC45A4 RAB3D MRPS7 PLEC RAB44 PLPPR2 CLSTN1 PARP10 MRPS23 SLC25A19 GGA3 GRINA GRB2 TNPO3 PGPEP1 SSBP4 MIF4GD IKZF1*	null

### MS Risk SNPs Are Enriched in VSE Regions of THP-1 Cells After 1,25(OH)_2_D_3_ Stimulation

The known MS risk SNPs have previously been found to be enriched in classic SE regions (identified by H3K27ac) of monocytes (3, 7). Therefore, we aimed to determine if MS risk SNPs are enriched in VSEs, as causal SNPs in these regions may have the potential to modulate the Vitamin D responsiveness of MS risk genes.

**Figure 4:**
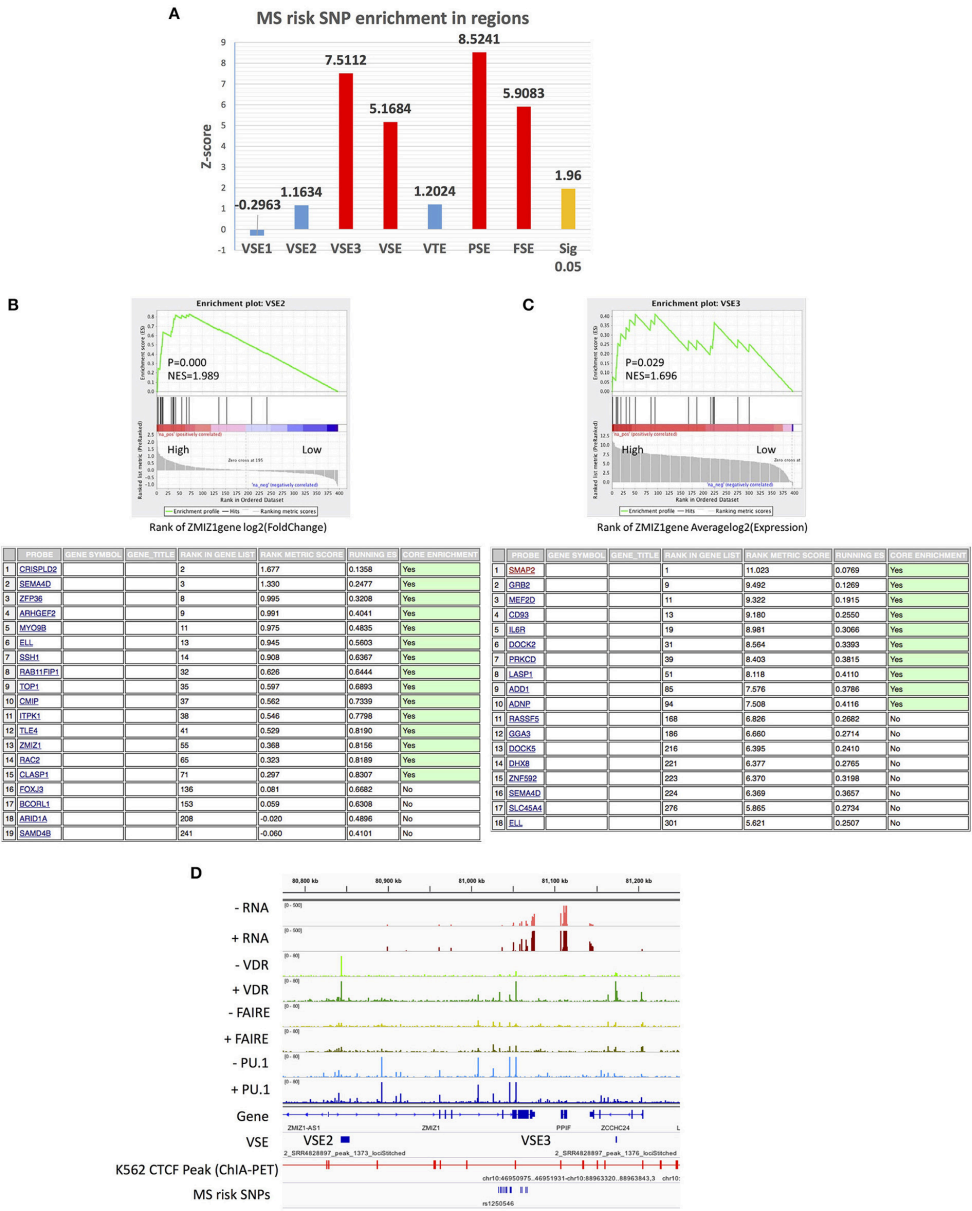
The association of MS risk SNPs and *ZMIZ1* gene set with VSE. **(A)** MS risk SNP enrichment in different SE regions. **(B)** VSE2-regulated genes are enriched in *ZMIZ1* positively associated genes with higher log_2_(fold change) (above); The names of these enriched VSE2-regulated genes (below). **(C)** VSE3-regulated genes are enriched in *ZMIZ1* positively associated genes with higher average log_2_(expression) (above); The names of these enriched VSE3-regulated genes (below). **(D)** Visualization of signal densities and VSE region around *ZMIZ1* gene.

Firstly, we analyzed the enrichment of known MS risk SNPs in both classic SE (identified by PU.1 peaks or FAIRE-seq peaks) and signal SE (VSE) regions after stimulation with 1,25(OH)_2_D_3_. We found that VSE regions are also enriched for MS risk SNPs with MS risk SNPs located in two VSE2 regions and two VSE3 regions (Figure 4A and Figures S3A–C). Interestingly, the genes near the VSEs (i.e., *UBASH3B, IRF8, PLEC, PARP10, GRINA*) all show stable high expression levels with only modest regulation by stimulation (Table 4 and Figures S3B,C). For genes with a stable and high expression level, even modest changes in expression may lead to biological effects, such as the modest regulation of *MYC* and *ZMIZ1* by VDR (15, 47). Therefore, MS risk SNPs in VSE regions may affect the function of VSEs that maintain the high and stable expression of key genes rather than inducing significantly higher expression.

**Table 4:** The gene near SE regions that overlap with MS risk SNPs.

**SE region**		**Gene in THP-1 cells after 1,25(OH)2D3 stimulation**			**MS risk SNPs including SNPs in LD region**		
		**Gene**	**Log_**2**_FC**	**AveExpr**	**Coordinate (0-based)**	**dbSNP ID**	**RegulomeDB score**
VSE	VSE2	*UBASH3B*	−0.1253362	5.050095	chr11:122518524	rs6589939	3a
		*IRF8*	−0.1469718	9.341672	chr16:86005837	rs68143182	5
	VSE3	*PLEC*	−0.5071387	8.468479	chr8:145007946	rs6989119	2a
		*PARP10*	−0.2369997	7.492962	chr8:145013892	rs4073081	2a
		*GRINA*	0.108769	7.872264	chr8:145014731	rs112979447	2a
PSE		*SYK*	0.5603835	8.668792	chr9:93563535	rs290986	5
		*UBASH3B*	−0.1253362	5.050095	chr11:122527119	rs7129071	2a
FSE		*PLEC*	−0.5071387	8.468479	chr8:145007946	rs6989119	2a
		*PARP10*	−0.2369997	7.492962	chr8:145013892	rs4073081	2a
		*GRINA*	0.108769	7.872264	chr8:145014731	rs112979447	2a
		*DENND3*	−0.1418788	7.662943	chr8:142104943	rs4961252	5

Furthermore, almost all the risk SNPs located in SE regions (40 of 45 SNPs in VSE, 70 of 85 SNPs in PSE, 33 of 33 SNPs in FSE) have RegulomeDB scores providing at least some evidence for a functional role such as being an eQTL, having transcription factor (TF) binding, a matched TF motif or DNase I hypersensitivity. We list the SNPs with the top RegulomeDB score for each SE gene in Table 4. Interestingly, among VSE1–3, only VSE3 is enriched for MS risk SNPs (Figure 4A), which is consistent with its higher GC content and high expression level.

### The MS Risk *ZMIZ1* Gene Set With VDR Super-Enhancers

Booth and colleagues used cohorts to find MS risk genes that are expressed differently in whole blood between MS patients and healthy controls, and they found that *ZMIZ1* and *EOMES* are the most significant two, and that this result can be repeated in other cohorts (13, 15). The *ZMIZ1* gene is highly expressed in myeloid cells including THP-1 monocytes and is considered a risk gene for MS. The *ZMIZ1* gene set (defined as genes whose expression is positively correlated with that of *ZMIZ1*; see methods) is under-expressed in the blood of MS patients and has been proposed as a gene signature for MS (15). Importantly, *ZMIZ1* is also Vitamin D-responsive. Therefore, we tested the association between VSEs and *ZMIZ1* gene set in THP-1 cells. The *ZMIZ1* gene set was downloaded from the file of a publication by Fewings et al. (14, 15), consisting of the top 200 genes positively correlated with *ZMIZ1* expression from each of three cohorts (Supplementary File 1).

By GSEA enrichment analysis, a cluster of genes with VSE2 regions (*ZMIZ1* gene cluster 1) from the *ZMIZ1* gene set were found to be enriched in genes that show a substantial change after 1,25(OH)_2_D_3_ stimulation (“High” region by log_2_(fold change) in *ZMIZ1* gene set, Figure 4B). Under the same experimental conditions, a cluster of genes with VSE3 regions (*ZMIZ1* gene cluster 2) from the *ZMIZ1* gene set is enriched in genes that show a high expression level (“High” region by average log_2_(expression) in *ZMIZ1* gene set, Figure 4C).

Furthermore, *ZMIZ1* itself also contains a VSE2 and is near a VSE3 region (Figure 4D). Although the VSE2 and VSE3 around *ZMIZ1* do not overlap with MS risk SNPs, they may be connected with SNPs via loops shown by CTCF ChIA-PET data in K562 cells from the ENCODE database (Figure 4D). In addition, among the *ZMIZ1* gene set, *TOP1* has a VSE2 and is under significant regulation by 1,25(OH)_2_D_3._ It is near (298.8 kb from) a MS risk SNP rs6065333 (Figure S4A). *GRB2* and *DOCK2* have VSE3 regions and high expression levels. They are overlapped with and near (440 kb from) MS risk SNPs rs9900529 and rs11957313, respectively (Figures S4B,C).

**Figure 5:**
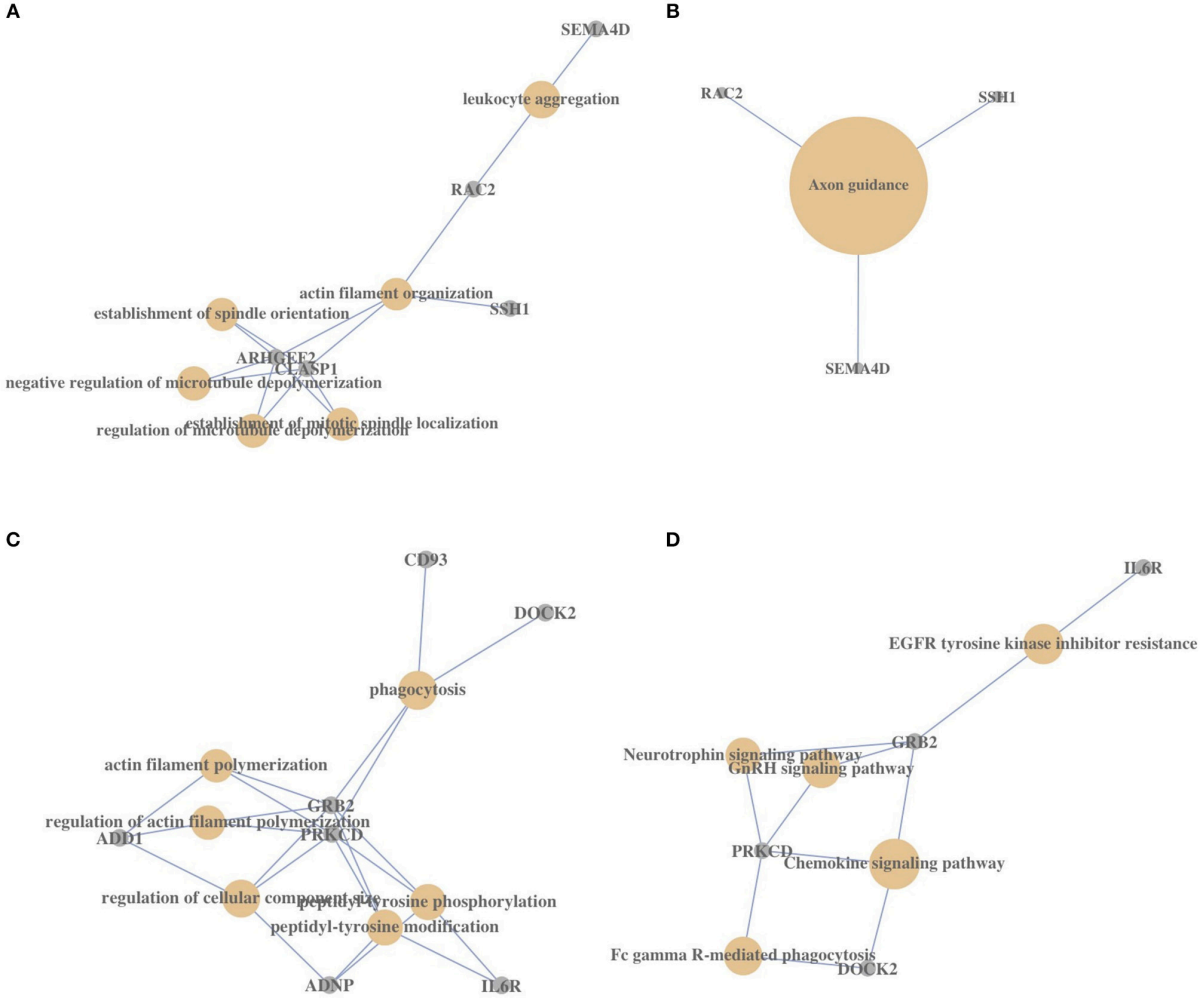
The biological process and signal pathway enrichment of *ZMIZ1*-associated gene clusters. **(A)** The GO BP terms enrichment for *ZMIZ1* gene cluster 1. **(B)** The KEGG signal pathway enrichment for *ZMIZ1* gene cluster 1. **(C)** The GO BP terms enrichment for ZMIZ1 gene cluster 2. **(D)** The KEGG signal pathway enrichment for ZMIZ1 gene cluster 2. Size of circle reflects the relative significance of *p*-value in each figure.

We also used GO biological process and KEGG pathway enrichment analysis to find potentially biologically important genes in the two clusters of genes that are associated with both VSE regions and MS risk. For *ZMIZ1* gene cluster 1, we found the genes *ARHGEF2* and *CLASP1* were enriched in five of the top six biological process terms. These enriched genes relate mainly to the functions of microtubule, spindle, and actin filament (Figure 5A). Among these biological process terms, the enrichment of “leukocyte aggregation” for genes *SEMA4D* and *RAC2* had the most significant *p*-value (*p* = 3.45 × 10^−5^). After KEGG pathway analysis, we found *SEMA4D, RAC2* and *SSH1* are also significantly (*p* = 4.13 × 10^−4^) enriched in “axon guidance” pathway (Figure 5B). These genes and their enriched terms/pathways are potentially under the regulation of Vitamin D via VSE2 regions and their impact on MS pathology has not yet been tested.

For *ZMIZ1* gene cluster 2, we found that the *PRKCD* and *GRB2* genes were included in all of the top six enriched biological process terms, which related mainly to the functions of actin filament, peptidyl-tyrosine, cellular component size and phagocytosis (Figure 5C). The enrichment of “phagocytosis” for genes *PRKCD, GRB*2, *CD93*, and *DOCK2* has the most significant *p*-value (*p* = 1.79 × 10^−5^). After KEGG pathway analysis, the enrichment of *PRKCD, GRB2*, and *DOCK2* in “chemokine signaling pathway” showed the most significance (*p* = 3.01 × 10^−4^) (Figure 5D). As mentioned above, the genes *GRB2* and *DOCK2* are also within the ± 500 kb region of MS risk SNPs (Figures S4B,C), and VSE regions around *IL6R, PRKCD* and *GRB2* are also enriched in the GO term: “positive regulation of immune system process” (Figure 1D and Supplementary File 1). These genes with a relative high expression level and their enriched terms/pathways are potentially under the regulation of Vitamin D via VSE3 regions and could be involved in the etiology of MS.

## Discussion

Much research has occurred around classic SE regions, but alterations of signal-induced SEs before and after stimulation have not been well explored. In addition, the relationship between many complex disease risk variants and environmental signal SEs have not been explored, including the interaction between Vitamin D induced VDR super-enhancers and MS risk SNPs in the etiology of MS. In this study, we used publicly available data (Table 1 and Table S1) to explore the features of VSE patterns and their regulatory effects on gene expression using *in silico* methods, in order to interrogate the role of the environmental factor Vitamin D on MS. Additional *in vitro* or *in vivo* evidence will be required to confirm the links between VSEs and MS risk proposed here.

We particularly focused on the role of 1,25(OH)_2_D_3_-induced SEs describing the distinct regions of VSEs compared with those of PSEs and FSEs (Figure 1), and then classified VSEs into three patterns: VSE1–3 (Figures 2A,B). VSE1–3 have different characteristics including their association with the densities of PU.1 binding and FAIRE-seq, the gene sets they regulate, GC content, and gene expression level (Figures 2C, 3A,C–E). We identified the genes with VSE1–3 regions that are also under significant regulation of 1,25(OH)_2_D_3_ (Figures 3A,B and Figure S1). We also showed that MS risk SNPs are enriched in VSE regions, especially the VSE3 pattern of VDR binding (Figure 4A and Figure S3A). This suggests that the potential causal genes in the regions defined by SNP association could be the ones that have VSE regions (Tables 3, 4 and Figures S2, S3B,C). Moreover, we found that two clusters of genes with VSE2 or VSE3 patterns are enriched in the significantly regulated genes or highly expressed genes of the *ZMIZ1* gene set (Figures 4B,C and Figure S4). The genes we identified in this study may be key points in the interaction between the environmental factor Vitamin D and genetic risk variants for the etiology and process of MS, and need further exploration with specific experiments designed to assess the role of the genetic variation in modulating Vitamin D regulated gene expression.

FAIRE-seq, PU.1 ChIP-seq and VDR ChIP-seq have all been used to capture enhancer regions in different cell types (48–50). However, their super-enhancer regions in one cell type have not been explored before. Our results showing distinct regions of VSE, PSE, and FSE, is consistent with one previous study showing that ERα, FoxA1 and AP2γ form different SE regions in MCF-7 cells (8). However, in another study (BioRxiv: http://dx.doi.org/10.1101/105262), the SEs formed by TGF-β signal terminal TF SMAD3 overlap a high proportion of the SEs formed by MED1 in mESC (73%) or in pro-B cells (64%). The research suggests that classic (MED1) SEs can provide a platform for signaling terminal TFs (SMAD3) to bind with a SE dense assembly, although there are still a large proportion (~60%) of SMAD3 SEs outside MED1 SE regions. Therefore, we hypothesize that the signal SE and classic SE regions do not overlap by a large proportion and are responsible for regulating different functional genes as reported here for VDR and PU.1 in THP-1 monocytes. However, if an external signal is important for cell differentiation and identity, its terminal TF signal SE will overlap the majority of classic SEs, in order to affect the expression of cell identity genes in response to the signal, as seen with SMAD3 and MED1 in mESCs.

A previous studying investigating ERα SEs (8) indicated that persistent ERα binding that existed both before and after estrogen stimulation, initiated the generation of ERα SE regions after stimulation. However, not all persistent signal TF ERα bindings induce secondary ERα binding sites around them to form SE regions after stimulation, and there are still some secondary ERα binding sites that form SE regions independent of persistent TF binding. Furthermore, there have been some studies showing that primary (or transient), persistent, and secondary signal TF binding have different characteristics. For example, persistent signal TF binding sites have more canonical motif enrichment (8, 16). In addition, by machine learning approaches one study found that different combinations of transient and persistent VDR binding sites in topologically associating domains (TAD) regulate different biological processes (16). As one TAD may include multiple SE regions with different generation mechanisms, here we focused on the SE regions [potential sub-TAD regions (51)] and only analyzed their combination of persistent and secondary signal TF binding.

We classified VSEs into three patterns based on the pattern of persistent or secondary VDR binding following stimulation with 1,25(OH)_2_D_3_. While VSE2 and VSE3 patterns are correlated with the signal density of PU.1 and FAIRE-seq peaks, VSE1 regions are not. All three VSE patterns have significantly higher regulatory effect on gene expression compared with VDR promoters and VDR typical enhancers, but VSE3 has the highest level of gene expression compared with other VDR binding regions.

Interestingly, we find that the ratio between the number of VSE1–3 regions is signal-specific, i.e., different signals (e.g., Vitamin D, estrogen, or glucocorticoid) have different ratios between the numbers of their signal SE1–3 (Table 2), potentially corresponding to the different genomic functions (e.g., pioneer TF, master TF, or just signal TF) of their terminal TFs (e.g., VDR, ERα, GR). The persistent VDR binding regions are ligand-insensitive, and exist before signal stimulation. The effects of VSE1 and VSE2 on gene expression after signal stimulation reflect a transcriptional memory, which is similar to the control of ligand-insensitive nuclear receptor PPARγ on the processive macrophage polarization (52).

By connecting gene expression levels with VSE regions and MS risk SNPs, we identified five significant VSE-regulated MS risk genes: *DENND6B, USP2, ASAP2, SEMA6B*, and *LRG1*. Among them, *DENND6B* is highly expressed in the brain (53), and its protein interacts directly with Rab GTPases involved in vesicle trafficking and cytokine production during the process of neuroinflammation (54, 55). USP2, a de-ubiquitinating enzyme, can regulate lipoprotein clearance by promoting deubiquitinylation and preventing the degradation of low-density lipoprotein receptor in HEK293T cells (56). In HL-60 macrophages, USP2 can regulate LPS-induced production of pro-inflammatory cytokines by reducing the polyubiquitination of octamer-binding transcription factor (Oct)-1 (57). ASAP2 can modulate Fc gamma receptor-mediated phagocytosis and cell migration, which are potentially associated with efferocytosis-mediated inflammation resolution and monocyte migration via the blood-brain barrier (58, 59). SEMA6B is associated with the signal pathway of “axon guidance” that is involved in both peripheral and central nervous system development, the disorder of which potentially impacts the pathology of MS. In particular, some semaphorins, as immune modulators, such as SEMA4D, are involved in the immune response by regulating immune cell–cell contacts and cell migration (60). In this study, we show that these MS risk genes are all highly expressed and significantly regulated by 1,25(OH)_2_D_3_ in THP-1 monocytes.

We also identified another five important MS risk genes with VSEs overlapping with MS risk SNPs: *UBASH3B, IRF8, PLEC, PARP10*, and *GRINA*. These genes are only modestly regulated by 1,25(OH)_2_D_3_. However, modest regulation cannot rule out their importance, especially when the genes have a key biological function. Among them, UBASH3B has a protein tyrosine phosphatase activity, which involves in the down-regulation and degradation of receptor-type tyrosine kinases, and promotes the accumulation of T cell receptors (61–63). The MS risk gene *IRF8* codes for an important transcription factor that is associated with chronic inflammation and binds the upstream regulatory region of type I IFN and IFN-inducible MHC class I genes (64). MS risk SNP rs35929052 that is associated with conversion to MS and disease relapse is close to *IRF8* (65). The expression of *PLEC* is changed by approximately 1.5-fold after 1,25(OH)_2_D_3_ stimulation in THP-1 monocytes. The related pathway of PLEC is “cytoskeletal signaling,” which is also associated with cell migration and phagocytosis (www.genecards.org) (66).

Finally, for analyzing the potential function of *ZMIZ1*-associated genes, GO ontology and KEGG pathway analysis were used. *ARHGEF2, RAC2, SEMA4D, PRKCD, GRB2*, and *DOCK2* were repeatedly enriched in important GO BP terms or KEGG pathways, such as “axon guidance,” “phagocytosis,” and “chemokine signaling,” which are all associated with the pathology of MS and MS process. Importantly, these genes are all Vitamin D-regulated and MS-associated.

Since VSE2 regions are formed based on pre-existing VDR binding sites, the cell functions enriched for *ZMIZ1* gene cluster 1, such as “leukocyte aggregation,” “actin filament organization,” and “axon guidance,” are potentially predetermined to be regulated by 1,25(OH)_2_D_3_ through forming VSE2 regions after stimulation. Therefore, VSE2 formation will increase the basal low-level expression of *ZMIZ1* gene cluster 1 and promote its associated functions after 1,25(OH)_2_D_3_ stimulation.

Because VSE3s are more active regulatory regions with the highest GC content, and the genes with VSE3 regions have the highest expression levels, it is not surprising that *ZMIZ1* cluster 2 genes with VSE3 regions are enriched in highly expressed genes. The cell functions enriched for *ZMIZ1* gene cluster 2, such as “phagocytosis” and “chemokine signaling,” are potentially constitutively active in THP-1 monocytes. VSE3 formation will sustain the basal high-level expression of *ZMIZ1* gene cluster 2 and reinforce its functions after 1,25(OH)_2_D_3_ stimulation.

In summary, our results support the importance of signal-induced nuclear receptor SEs in the signal stimulation process, and detail the characteristics of signal SEs by classifying 1,25(OH)_2_D_3_-induced vitamin D receptor SE regions into three patterns. Importantly, we connect MS risk SNPs with Vitamin D downstream VSE regions for the first time.

In future research, the actions of signal SEs should be an important consideration parallel to classic SEs, which could provide more information about cell status with signal perturbations and deeper insight into the regulatory mechanisms of signal dependent transcription factors (e.g., nuclear receptor, STATs, Smad3) in the genome. The understanding of signal SEs can help elucidate the interaction between environmental risk factors and genetic factors in the onset and progression of complex diseases by allowing us to explore the interaction between environmental signals and the function/identity of causal cell-types. Important VSE-associated MS risk genes in monocytes were predicted, whose normal functions may be disturbed by MS risk alleles around the VSE regions. These genes that are associated with both VSE and MS risk regions warrant further analysis to elucidate the mechanisms involved.

## Data Availability

Publicly available datasets were analyzed in this study. This data can be found here: https://www.ncbi.nlm.nih.gov/geo/query/acc.cgi?acc=GSE89431 (and GSE89178/GSE69303/GSE27437/GSE60270/GSE25021/GSE71616/GSE109131).

## Author Contributions

ML: designed the study, acquired data, analyzed data, and wrote the manuscript. BJM, KPB, BVT and HK: edit the manuscript. ML and HK: conception of the project.

### Conflict of Interest Statement

The authors declare that the research was conducted in the absence of any commercial or financial relationships that could be construed as a potential conflict of interest.

## Supplementary Material

Figure S1 The genes that are significantly regulated by VSE1–3. The genes that are significantly regulated in the genome-wide MDplot from RNA-seq data. Blue words: long non-coding RNA **(Left)**. The genes that are significantly regulated in the VSE finding curve **(Right)**.

Figure S2 The representative genomic regions of the genes with VSEs that are associated with MS risk SNPs (bold in Table 3). **(A)** *DENND6B*. **(B)** *USP2*. **(C)** *ASAP2*. **(D)** *LRG1* and *SEMA6B*.

Figure S3 The association between MS risk SNP and VSE1-3 region. **(A)** MS risk SNPs enrichment in different SE regions. The permutation figure showed the α = 0.05 expected values (red line) from 1,000 permutations and observed values (green line). **(B)** The genomic regions of the genes with VSEs that are overlapped with MS risk SNPs. **(C)** The genomic regions of the genes with PSE or FSE that are overlapped with MS risk SNPs. VSE, VDR SE; PSE, PU.1 SE; FSE, FAIRE SE.

Figure S4 The representative genomic regions of the *ZMIZ1*-associated genes with VSEs that are near MS risk SNPs. **(A)** *TOP1*. **(B)** *GRB2*. **(C)** *DOCK2*.

Table S1 Control and hormone-stimulated ChIP-seq data.

## Abbreviations

BMDM: bone marrow-derived macrophages
ChIA-PET: chromatin interaction analysis by paired-end tag sequencing
ChIP-seq: chromatin immunoprecipitation sequencing
CTCF: CCCTC-binding factor
dex: dexamethasone
DHS: DNase hypersensitivity sites
D3: 1,25(OH)_2_D_3_
ECC-1: endometrial cancer cell line
eQTL: expression quantitative trait loci
GR: glucocorticoid receptor
GRSE: super-enhancer formed by GR binding regions
ERα: estrogen receptor α
ER SE: super-enhancer formed by ERα binding regions
E2: estradiol
FAIRE: formaldehyde-assisted isolation of regulatory elements
FSE: super-enhancer formed by FAIRE regions
GEO: gene expression omnibus
GM10855/GM10861: human lymphoblastoid cells
GWAS: genome wide association studies
LD: linkage disequilibrium
LS180: human colorectal cancer cells
LX2: human hepatic stellate cells
MED1: mediator complex subunit 1
MCF-7: human breast cancer cells
mESC: murine embryonic stem cells
MS: multiple sclerosis
NGS: next generation sequencing
PSE: super-enhancer formed by PU.1 binding regions
RS4;11: human acute leukemia cells (B cell and monocyte like)
SE: super-enhancer
SNP: single nucleotide polymorphism
TAD: topologically associating domain
TE: typical enhancer
TF: transcription factor
THP-1: human monocyte-like cells
VDR: Vitamin D receptor
VSE: super-enhancer formed by VDR binding regions
VTE: typical enhancer formed by VDR binding.
