# Supplementary figures and images for: The Association Between Vitamin D and Multiple Sclerosis Risk: 1,25(OH)2D3 Induces Super-Enhancers Bound by VDR
Source: Front Immunol. 2019 Mar 19;10:488. doi: 10.3389/fimmu.2019.00488 (PMC6433938; doi:10.3389/fimmu.2019.00488)

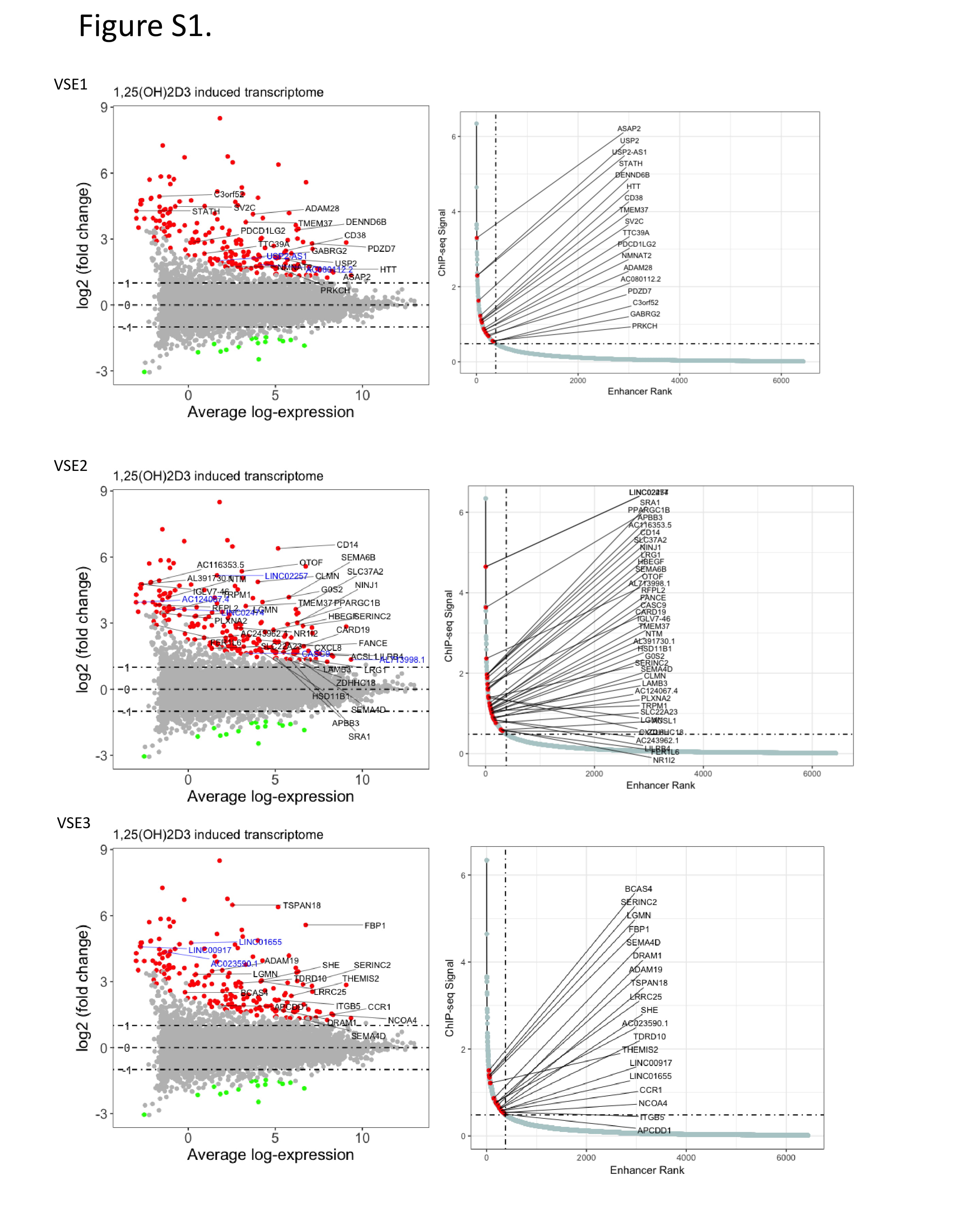

Supplement: Figure S1 — The genes that are significantly regulated by VSE1–3. The genes that are significantly regulated in the genome-wide MDplot from RNA-seq data. Blue words: long non-coding RNA (Left). The genes that are significantly regulated in the VSE finding curve (Right). [file Image_1.JPEG]

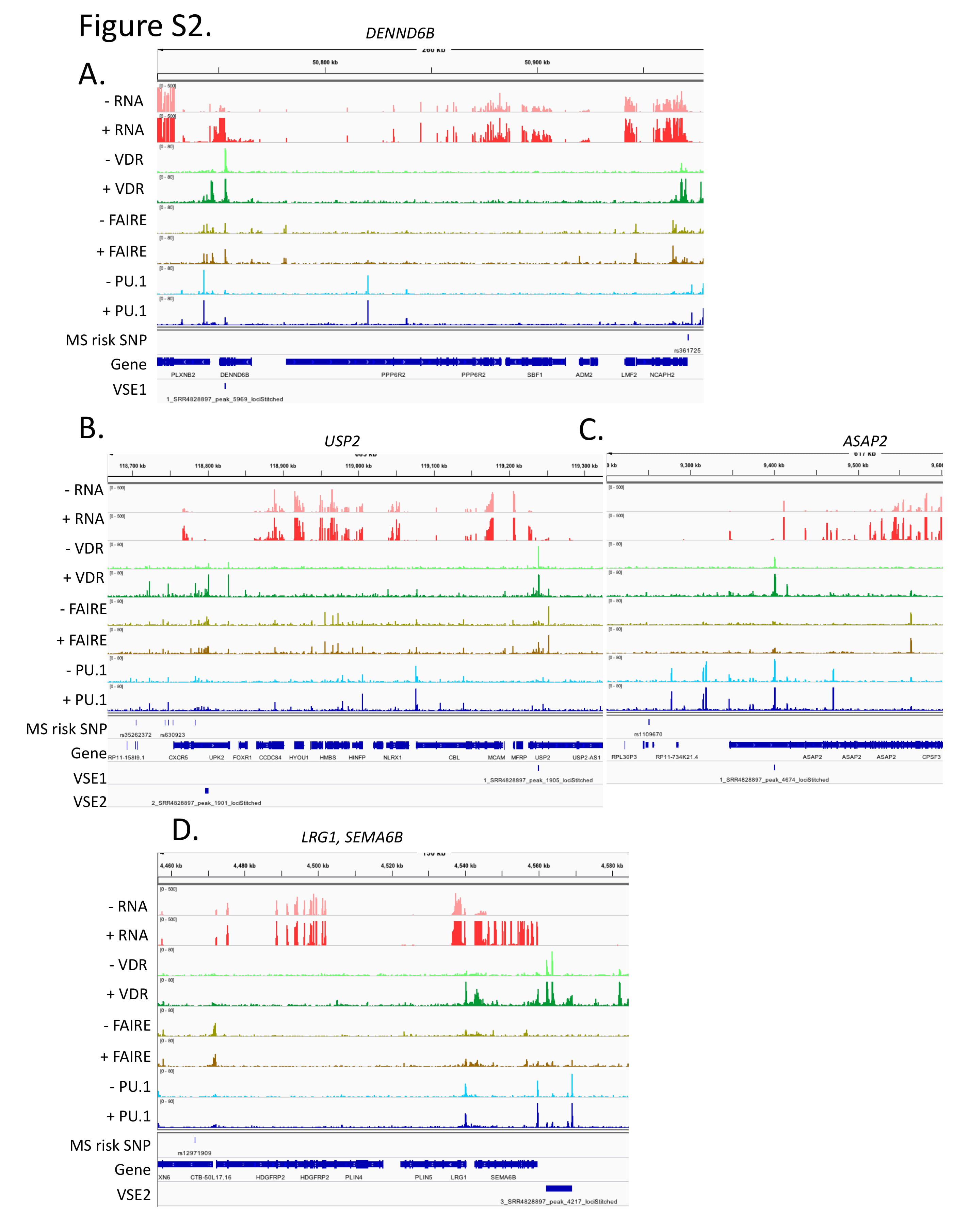

Supplement: Figure S2 — The representative genomic regions of the genes with VSEs that are associated with MS risk SNPs (bold in Table 3). (A) DENND6B. (B) USP2. (C) ASAP2. (D) LRG1 and SEMA6B. [file Image_2.JPEG]

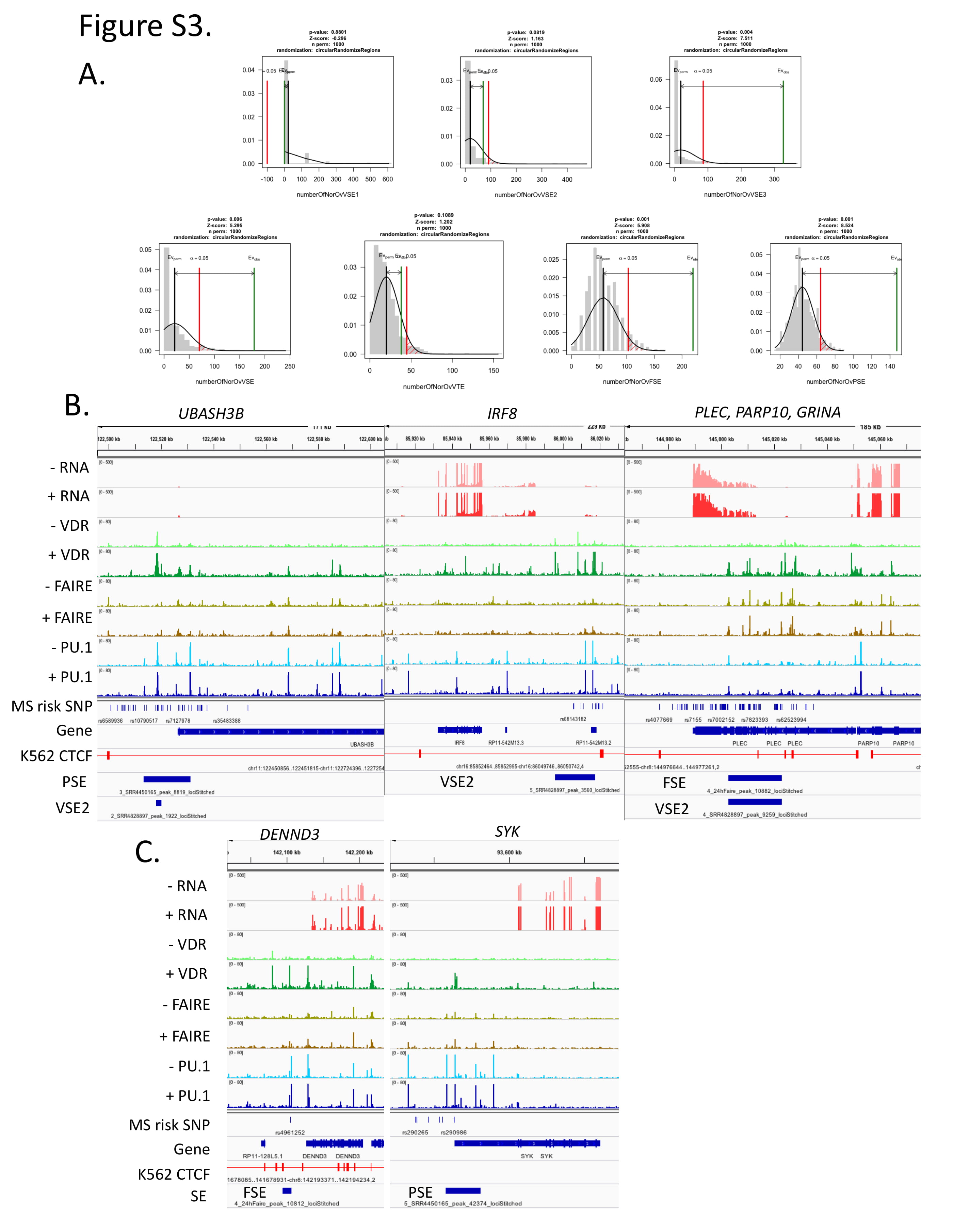

Supplement: Figure S3 — The association between MS risk SNP and VSE1-3 region. (A) MS risk SNPs enrichment in different SE regions. The permutation figure showed the α = 0.05 expected values (red line) from 1,000 permutations and observed values (green line). (B) The genomic regions of the genes with VSEs that are overlapped with MS risk SNPs. (C) The genomic regions of the genes with PSE or FSE that are overlapped with MS risk SNPs. VSE, VDR SE; PSE, PU.1 SE; FSE, FAIRE SE. [file Image_3.JPEG]

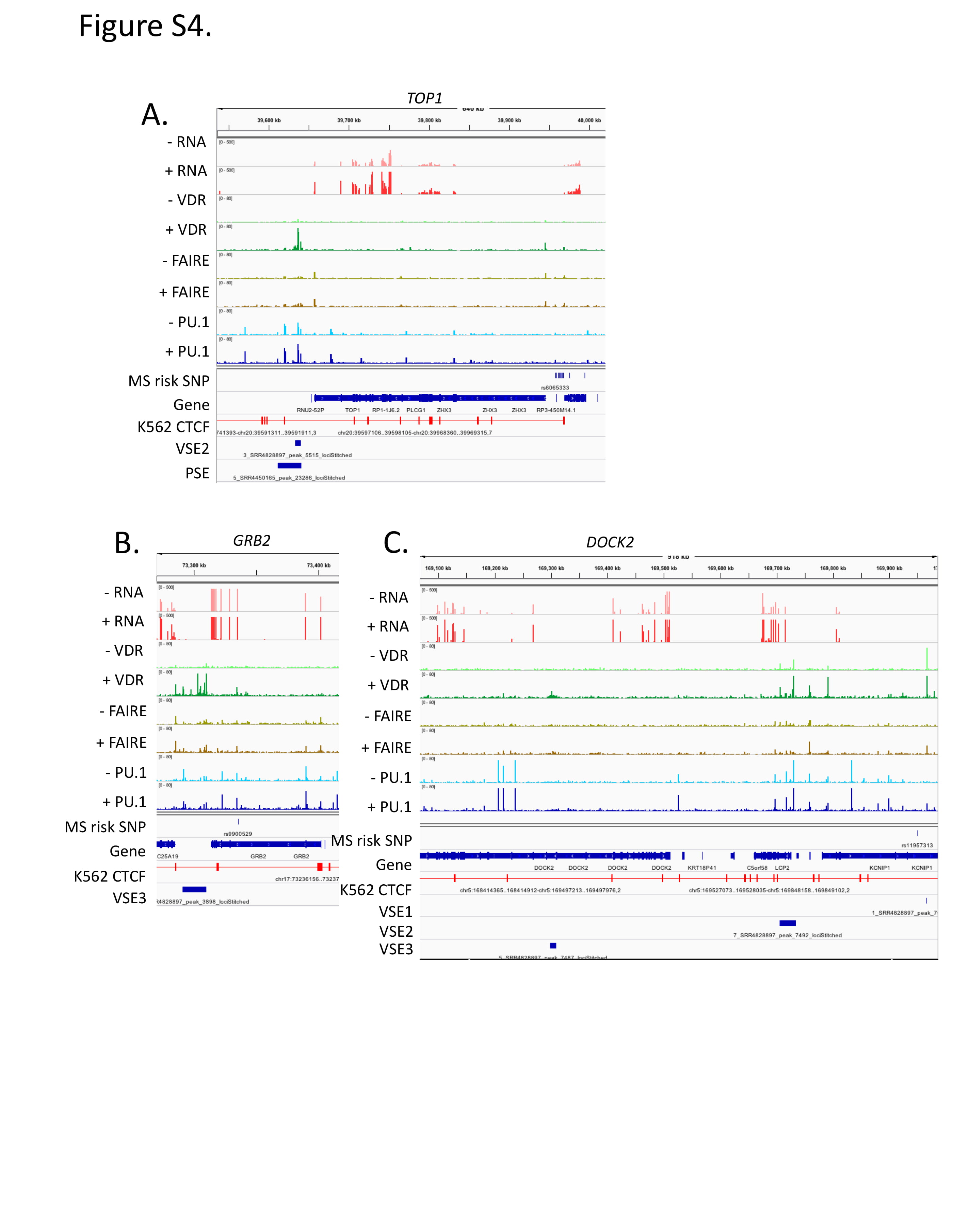

Supplement: Figure S4 — The representative genomic regions of the ZMIZ1-associated genes with VSEs that are near MS risk SNPs. (A) TOP1. (B) GRB2. (C) DOCK2. [file Image_4.JPEG]
